# Correlation between Liver fat Content Determined by Ultrasonic Attenuation Imaging and Lipid Metabolism in Patients with Non-Alcoholic Fatty Liver Disease

**DOI:** 10.2174/0115734056335310250217064323

**Published:** 2025-03-17

**Authors:** Yanhong Hao, Yanjing Zhang, Guolin Yin, Lei Zhang, Liping Liu

**Affiliations:** 1 Department of Ultrasonography, First Hospital of Shanxi Medical University, Taiyuan, Shanxi, 030001, China; 2 Department of Interventional Ultrasound, First Hospital of Shanxi Medical University, Taiyuan, Shanxi, 030001, China; 3 Department of Magnetic Resonance, First Hospital of Shanxi Medical University, Taiyuan, Shanxi, 030001, China

**Keywords:** Non-alcoholic fatty liver disease, Attenuation imaging, Type 2 diabetes, Ultrasonic attenuation imaging, Intraclass correlation coefficient, Proton density fat fraction

## Abstract

**Objective:**

This study aimed to investigate the utility of ultrasonic attenuation imaging (ATI) in assessing the relationship between hepatic fat content and lipid metabolism in patients with type 2 diabetes mellitus (T2DM).

**Methods:**

239 patients diagnosed with T2DM were included, with liver fat quantified using proton density fat fraction (PDFF). We analyzed the variance in ATI across various grades of fatty liver and its correlation with clinical parameters. Additionally, a receiver operating characteristic curve (ROC) was employed to evaluate the diagnostic accuracy of ATI for different degrees of fatty liver, determining optimal diagnostic thresholds while calculating sensitivity and specificity. Furthermore, we assessed the reliability of ATI and SWE in measuring liver acoustic attenuation and elastic stiffness using the intraclass correlation coefficient (ICC).

**Results:**

We observed significant variations in ATI across different grades of fatty liver (*p*<0.001). ATI exhibited positive correlations with SWE, BMI, GLU (OH), steatosis grade, ALT, TG, and UA, while demonstrating a negative correlation with HDL-c. Notably, the correlation coefficient with steatosis grade was 0.784, indicating a strong association. The equation for the stepwise multiple linear regression model used is as follows: ATI=0.338+0.014×TG+0.052×BMI+0.001×ALT+0.113×SWE. AUROCs indicated the best cutoffs for ATI in different degrees of steatosis to be as follows: ≥ S1 = 0.665 dB·cm-1·MHz-1 (AUC = 0.899); ≥ S2 = 0.695 dB·cm-1·MHz-1 (AUC = 0.939); ≥ S3 = 0.745 dB·cm-1·MHz-1 (AUC = 0.937). The ICC values for ATI and SWE in liver-mimicking measurements exceeded 0.75 (*p*<0.001), signifying excellent repeatability.

**Conclusion:**

The ATI could quantitatively assess the severity of fatty liver, enabling effective identification of patients suitable for liver biopsy referral.

## 1 INTRODUCTION

Non-alcoholic fatty liver disease (NAFLD) constitutes a metabolic stress-induced hepatic injury intricately linked to both insulin resistance and genetic predisposition [1]. Type 2 diabetes mellitus (T2DM) is a significant risk factor contributing to the rapid progression of NAFLD [2]. The incidence of NAFLD in cases of obesity and metabolic syndrome surpasses that of the general population, highlighting a concerning upward trend [3, 4]. Prolonged elevation in inflammatory mediators within the fatty liver milieu poses a significant risk, potentially leading to liver fibrosis, cirrhosis, or hepatocellular carcinoma [5, 6]. Currently, the global prevalence of NAFLD stands at a noteworthy 25%, indicating a substantial risk of chronic liver disease [7]. Within Europe and the United States, NAFLD has become a primary driver for liver transplantation [8]. Elevated hepatic fat levels accelerate disease progression. Consequently, the timely identification of hepatic steatosis, coupled with proactive intervention, assumes paramount significance in ameliorating the severity of fatty liver and halting disease advancement [9]. The gold standard for non-invasive quantification of liver fat within imaging modalities is the proton density fat fraction (PDFF) [10, 11]. Unfortunately, routine implementation of PDFF encounters impediments due to its prohibitive cost and suboptimal patient acceptance. Recent investigations, however, have revealed a notable correlation between ultrasonic attenuation imaging (ATI) and both the extent and grade of steatosis [12]. In light of the close relationship among NAFLD, insulin resistance, and genetic susceptibility, our study emphasizes the correlation of ATI with the degree of hepatic steatosis and early liver fibrosis in the T2DM population, providing a basis for the early prediction and management of NAFLD in the T2DM population.

This study aimed to elucidate the utility of ATI in the quantitative diagnosis of fatty liver in individuals with T2DM-associated NAFLD, while concurrently examining its correlation with clinically pertinent parameters.

## 2 MATERIALS AND METHODS

### 2.1 Participants

All individuals included in this investigation were patients diagnosed with T2DM at our medical institution between January 2021 and October 2022, adhering to predefined inclusion and exclusion criteria as previously outlined [13]. To ensure optimal ultrasound imagery, we selectively excluded individuals with a body mass index (BMI) exceeding 35 [14] and a skin-to-liver capsule distance greater than 2.5 cm [15]. Ultimately, 232 subjects were admitted out of the initially identified pool of 239 patients. Concurrently, during the same time frame, a cohort of 50 robust and healthy subjects served as the control group for the assessment of ultrasonic ATI and SWE. Inclusion criteria for these normative subjects included an absence of prior liver afflictions, the absence of hepatic steatosis or focal nodules, intact liver functionality, and the absence of metabolic disorders, hypertension, as well as other chronic conditions, including cardiovascular and cerebro-vascular ailments. PDFF, ascertained through a multi-echo Dixon sequence conducted within one week preceding the ultrasound examination, was used to quantify hepatic fat content. Categorization was based on a PDFF threshold of 5.0% or below classified as the negative group (S0), 5.0%-10.0% as the mild group (S1), 10%-16.37% as the moderate group (S2), and 16.37%-23.3% as the severe group (S3) [16].

This study adhered to the principles outlined in the 2013 Helsinki Declaration, and all participants provided their consent by signing the informed consent form approved by the Committee of the First Hospital of Shanxi Medical University (approval no. K-K0139).

### 2.2 Study Design and Setting

#### 2.2.1 Ultrasonic Instruments

For this investigation, a Canon Aplio i800 chromatic ultrasonic diagnostic apparatus was utilized. It features a probe frequency range of 1-8 MHz, a dynamic range of 60 dB, and a frame rate of 30 fps.

### 2.3 Methods

After fasting for a minimum of 6 hours, patients were positioned supine with their upper limbs elevated laterally to the head. The ultrasonic probe was then carefully placed over the intercostal space, reaching a depth of 12-14 cm for scanning. Patients were instructed to maintain a calm breathing state while briefly holding their breath. The instrument was subsequently switched to the ATI mode. The sampling frame, positioned 1-2 cm from the liver capsule, meticulously avoided major blood vessels, with dimensions fixed at 3×3 cm. ATI measurements were taken from a relatively avascular section of the liver parenchyma under conventional ultrasound guidance. The resulting average value was derived from 5 consecutive measurements, meeting an image quality criterion of *r*2 > 0.90.

**Fig. (1):**
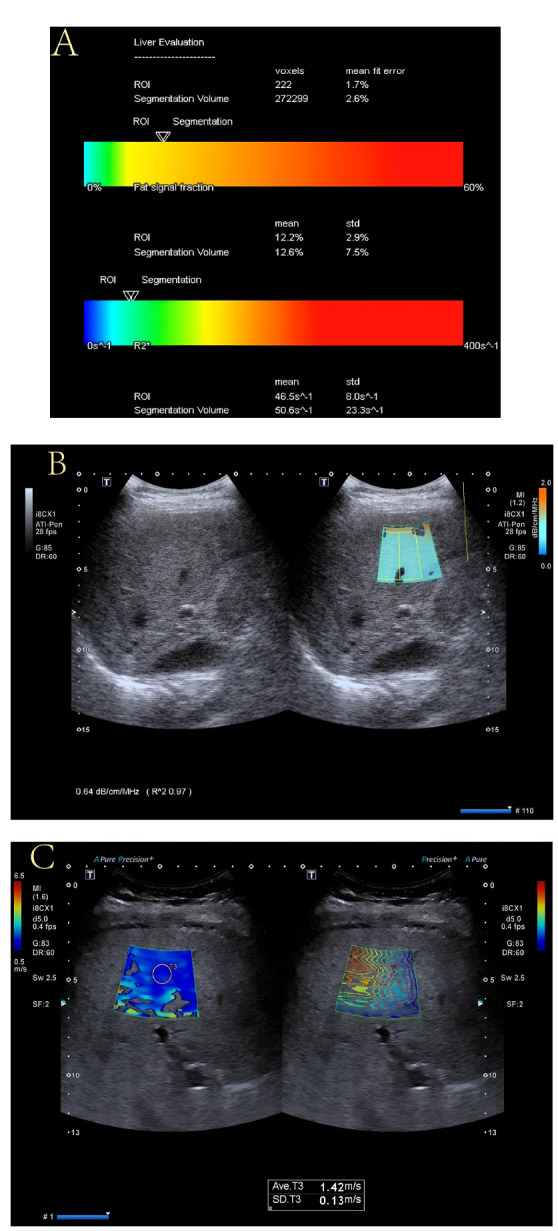
ATI and SWE maps of mild fatty liver. **A**: FF value was 12.2%, suggesting the patient to have a mild fatty liver. **B**. On the measurement of ultrasound attenuation imaging, the ATI was 0.79 dB/cm/MHz. **C**. On the measurement of elastic shear wave imaging, the SWE was 1.42 m/s.

After transitioning to shear wave elastography (SWE) mode, liver stiffness was assessed at the same position. The quality control image displayed a coherent representation during consistent sound wave propagation, with the sampling volume consistently set at 1.0 cm to measure the liver's elastic stiffness parameter (Fig. **1**). Importantly, all examinations were conducted by two ultrasound practitioners, each with over a decade of professional experience. The consistency and standardization of measurements among operators were ensured through training or the use of standardized operating procedures.

### 2.4 Data Sources/Measurement

#### 2.4.1 Clinical and Laboratory Indicators

In line with previous research findings, detailed patient demographic parameters following admission, including gender, age, BMI, and various assay indicators, were meticulously recorded. Fasting venous blood samples were systematically collected from the entire patient cohort. Biochemical indices were assayed using the AU5800 system in Japan as follows: alanine aminotransferase (ALT) and aspartate aminotransferase (AST) using the rate method; total protein (TP) *via* the biuret method; albumin (ALB) employing the bromocresol green method; globulin (GLB) using the TP-Alb method; total cholesterol (TC) utilizing the enzyme method; triglycerides (TG) through the GPO-POD method; high-density lipoprotein cholesterol (HDL-C) and low-density lipoprotein cholesterol (LDL-C) employing the direct method; uric acid (UA) ascertained *via* the urokinase peroxidase method; glucose metabolism indices analyzed using the Roche Cobas 8000 and Roche Cobas 6000 systems; glucose *via* the hexokinase method; C-peptide (0h) using the chemiluminescence method; and glycated hemoglobin (HbA1c) determined by the immunoturbidimetric method. All outcomes were acquired expeditiously within a strict 2-hour post-sampling timeframe.

### 2.5 Body-mimicking Measurement

To ensure the reproducibility of ultrasound detection, simulative materials in the form of zinc alkane gel (sourced from the Institute of Acoustics, Chinese Academy of Sciences, and calibrated on November 11, 2022) were utilized. These mimics were characterized by predetermined acoustic attenuation values (0.31, 0.69, and 1.02 dB cm-1 MHz-1) and established hardness values (3.7, 13, 25, and 50 Kpa). Following meticulous selection, both ATI and SWE technique measurements were assessed by two skilled ultrasonographers, as depicted in Fig. (**2**).

### 2.6 Statistical Methods

SPSS version 23.0, a statistical analysis software application, and Free Statistics (version 2.0) were utilized for meticulous data processing. Normal distribution variables have been reported as mean ± standard deviation (SD), whereas variables with skewness have been reported as median interquartile range (IQR). The frequency and percentage (%) representations of categorical variables were used. Group comparisons were performed using the x^2^ test and analysis of variance. In cases where data exhibited non-homogeneity of variance, the rank sum test was employed. Following analysis of variance, post-hoc comparisons were conducted using the LSD method. Pearson and Spearman correlation analyses were employed to examine the relationship between ultrasonic ATI and clinical laboratory parameters. Selected indicators were then incorporated into a stepwise multiple linear regression analysis to construct a model encapsulating ATI-related parameters. The diagnostic efficacy of ATI across various degrees of fatty liver was evaluated using a ROC curve. The reproducibility of measurements among different operators was assessed using the ICC.

## 3 RESULTS

### 3.1 General Information Comparison

**Fig. (3):**
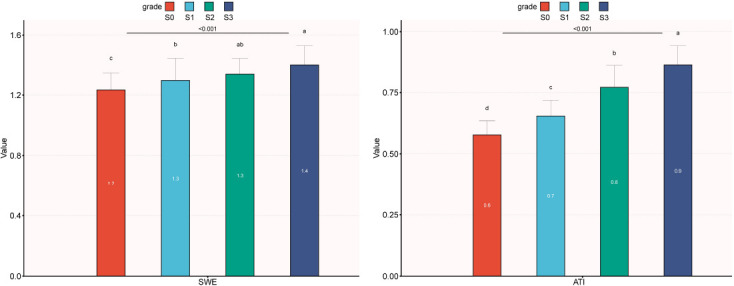
The relationship between ATI and liver steatosis grading. The ATI and SWE showed an increasing trend with an increase in fatty liver steatosis grade.

Ultimately, this study encompassed a total of 232 participants. The general characteristics of T2DM participants with different grades of steatosis in NAFLD are detailed in Table **1**. In the four different grades of hepatic steatosis, most laboratory indicators and ultrasound monitoring indicators show significant differences. A higher BMI is more likely to be associated with a higher degree of steatosis. As the degree of steatosis increases, fasting blood glucose, liver enzymes, TC, TG, HDL-C, and serum UA all increase, with a *p-*value < 0.05. With the progression of the degree of steatosis, both ATI and SWE significantly increase, aligning with the increasing grades of fatty liver. Ultrasonic ATI displayed distinct patterns across all groups, demonstrating an upward trend. SWE did not reveal significant differences between the S2 and S3 groups, yet substantial distinctions were evident among the remaining groups, indicating a rising trend (Fig. **3**). In contrast, gender, LDL-C, and HbA1c were not different among 4 different grades of steatosis in NAFLD.

### 3.2 Changes in ATI and SWE Across different HbA1c Stratifications

The S0 group was divided into three tiers based on HbA1c levels: below 7%, 7-9%, and exceeding 9%. A total of 60 cases with comprehensive data were analyzed. When compared to the healthy control group, significant variations were observed in SWE. However, within the S0 group, no significant differences in SWE were noted across different strata. ATI in the S0 group exhibited variability compared to the healthy control group across distinct stratifications. Specifically, when HbA1c stratification in the S0 group ranged from 7-9%, ATI in the S0 group displayed statistical variance from the healthy control group, although the difference in ATI within the S0 group was not statistically significant (Table **S1**).

### 3.3 Assessment of the Reproducibility of ATI and SWE

The ICCs for ATI (0.977 and 0.995) and SWE (0.995 and 0.998) conducted by different operators, in the context of graded fatty liver, all exceeded the threshold of 0.75, as shown in Table **2**.

### 3.4 Correlation Analysis of ATI with Laboratory Indices and Fatty Liver Grade

Further research indicates that ATI is significantly positively correlated with SWE, BMI, GLU, the degree of fatty liver, ALT, TG, and UA, with correlation coefficients of 0.363, 0.409, 0.202, 0.784, 0.290, 0.456, and 0.385, respectively (all *p*-value < 0.05). Conversely, a significant negative correlation was observed with HDL-C with a correlation coefficient of -0.254. These indicators were incorporated into a stepwise multiple linear regression model (α input = 0.05, α output = 0.10), resulting in a model of significance. The outcomes, as detailed in Table **3**, indicated a significant relationship, with *F* = 15.179, p < 0.001, R^2^ = 0.254, and *r*^2^ = 0.238 post-adjustment. According to the standardized coefficients, TG demonstrated the closest association with ATI, followed by BMI, ALT, and UA. The regression model equation for ATI, derived from stepwise multiple linear regression, is expressed as follows: ATI = 0.338 + 0.014×TG + 0.052×BMI + 0.001×ALT + 0.113×SWE (Table **4**).

### 3.5 Diagnostic Efficacy of ATI for different Degrees of Fatty Liver

**Fig. (4):**
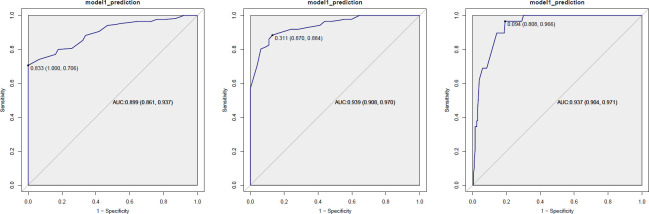
Receiver operating characteristic (ROC) curve of ATI for different steatosis grades. From left to right: steatosis grade ≥ S1, steatosis grade ≥ S2, steatosis grade ≥ S3.

A noticeable difference in ATI was apparent between the S0 group and fatty liver groups of different grades (*p* < 0.001), with ATI steadily increasing in the S1, S2, and S3 groups (*p* < 0.001) (Table **1**, Fig. **3**). The ROC curve highlighted the high diagnostic efficacy of ATI across various grades of fatty liver (Fig. **4** and Table **5**).

## 4 DISCUSSION

NAFLD has a high global prevalence [7]. Within the spectrum of NAFLD, NASH is a key link leading to the progression of liver fibrosis [17]. Additionally, according to epidemiological research, NAFLD and insulin resistance are interrelated; the coexistence of NAFLD and T2DM worsens the course of fibrillation [18-22]. There is a wealth of literature on non-invasive imaging assessment of NAFLD, while there are fewer studies on ATI, all of which have been conducted on the entire population or have not been performed in T2DM patients [23, 24]. Our study highlighted the correlation between ultrasound markers, such as ATI, and various laboratory indicators and liver fibrosis in NAFLD among T2DM patients. The specific parameter characteristics are provided in Table **1**.

PDFF has excellent diagnostic value for assessing liver fat content and providing the classification of histological steatosis, even surpassing the limitations of liver biopsy sampling [25, 26]. Vibration-controlled transient elastography (VCTE) is widely used for the diagnosis of NAFLD and predicting the occurrence of liver-related events associated with fatty liver [27, 28]. However, the diagnostic value of VCTE in grading fatty liver, especially in moderate to severe fatty liver, is limited [29]. Recent studies have shown a significant correlation between PDFF and ATI [30, 31]; in particular, the accuracy of diagnosing mild to moderate hepatic steatosis of ATI is the same as PDFF [32]. The consistency and precision between ATI and steatosis grading, compared to liver biopsy outcomes, surpassed that of VCTE [33]. Our study used PDFF as the gold standard to confirm the strong correlation between ATI and different grades of NAFLD. We also found a positive correlation between ATI and SWE in this study, which implies that higher grades of steatosis, as quantified by ATI, may be indicative of more pronounced liver fibrosis.

The elevation of ATI is significantly positively correlated with the grading of fatty liver and SWE, which suggests that clinically, interventions for fatty liver can be conducted more accurately and promptly, thereby slowing down or interrupting the progression of liver fibrosis. Our study results supported the Consensus Recommendations from the British Association for the Study of the Liver (BASL) and British Society of Gastroenterology (BSG) NAFLD Special Interest Group, encouraging active assessment of high-risk patients for the presence of liver fibrosis in clinical practice. In addition, undertaking second-stage non-invasive monitoring in the community can lead to the early detection of severe liver disease [34].

More importantly, even in the S0 group of diabetic populations, SWE exhibited statistical variances compared to the normal healthy population [24]. After stratified analysis of glycated hemoglobin, there was a difference found between SWE in the S0 group and the healthy control group. This suggests that managing glycated hemoglobin levels may effectively slow the progression of liver fibrosis in diabetic patients. Therefore, ATI technology emerges as a promising tool in the non-invasive diagnosis of mild to moderate fatty liver.

Additionally, the study outcomes underscored the close association of TG, BMI, and ALT with ATI. The elevation in TG, BMI, and ALT levels in patients corresponded with an intensification of fatty liver severity, heightening the peril of liver fibrosis. Notably, patients with suboptimal fasting blood glucose control exhibited a markedly higher severity of fatty liver compared to those with normal fasting blood glucose. Consequently, mitigating fasting blood glucose levels through dietary and exercise interventions aimed at improving TG, BMI, and UA levels holds promise for diminishing the severity of NAFLD.

## 5 LIMITATIONS

While acknowledging the merits of this study, it is imperative to address its limitations. First, the gold standard for the diagnosis of fatty liver is histological diagnosis. Considering the feasibility in clinical practice, PDFF is the non-invasive diagnostic gold standard for assessing liver fat content, a standard that has been confirmed by multiple studies and promoted for clinical application [25]. Secondly, we recruited participants more likely to be at risk for fatty liver, which limits the generalizability of our study results to other populations. In future research, it is necessary to further expand the scope of the study population. Furthermore, the inclination of diabetic patients toward stringent dietary control and medication post-diagnosis likely contributed to a dearth of severe fatty liver cases in the study cohort. This result is consistent with the views presented in the literature published in The Lancet in 2022 [35]. Therefore, we need to conduct further large-sample studies in external populations to enhance the diagnostic and discriminative efficacy of ATI for severe fatty liver and liver fibrosis.

## CONCLUSION

In summary, ATI emerges as a valuable asset for the non-invasive assessment of hepatic steatosis levels among individuals with NAFLD and concurrent T2DM. When coupled with relevant clinical parameters, ATI demonstrates an enhanced capacity to accurately measure the extent of hepatic steatosis, making it suitable for precise quantitative surveillance in the ongoing assessment of fatty liver severity. Before it can be adopted as a non-invasive tool for the assessment of NAFLD, further prospective research focusing on severe fatty liver disease is needed.

## Figures and Tables

**Fig. (2):**
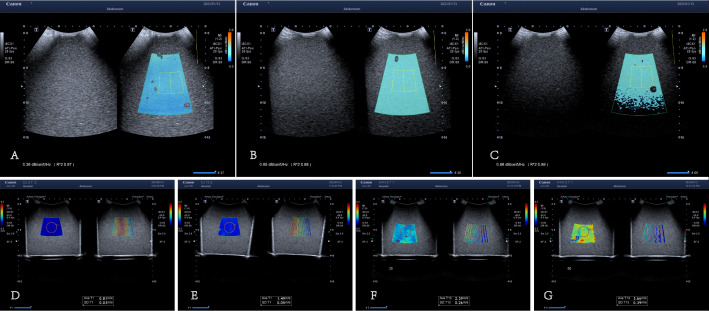
Different phantoms' ATI and SWE measurements. **A**-**C** represents the measurement image of ultrasonic attenuation. **A**. The rated ultrasonic attenuation value of the phantom is 0.31 cm-1·MHz-1, and the ultrasonic measurement value is 0.36 cm-1·MHz-1. **B**. The rated ultrasonic attenuation value of the phantom is 0.69 cm-1·MHz-1, and the ultrasonic measurement value is 0.65 cm-1·MHz-1. **C**. The rated ultrasonic attenuation value of the phantom is 1.02 cm-1·MHz-1, and the ultrasonic measurement value is 0.86 cm-1·MHz-1. **D**-**G** are the measurement images of ultrasonic elasticity. **D**. The rated hardness value is 3.7 kPa, and the SWE measured value is 0.81m/s. **E**. The rated hardness value is 13 kPa, and the SWE measured value is 1.49m/s. **F**. The rated hardness value is 25 kPa, and the SWE measured value is 2.39m/s. **G**. The rated hardness value is 50 kPa, and the SWE measured value is 3.66m/s

**Table 1:** Clinical data and comparison of ATI and SWE in groups with different grades of fatty liver.

	**S0**	**S1**	**S2**	**S3**	**F/χ^2^**	**P**
Sex	-	-	-	-	-	-
Male	34	55	35	18	1.714	0.634
Female	28	29	22	11	-	-
BMI (kg/m^2^)	-	-	-	-	-	-
<18.5	4	0	1	1	40.982	<0.001
18.5-24	44	40	13	3	-	-
>24	14	44	43	25	-	-
Age (y)	56.05 ± 11.44^a^	55.75 ± 11.69^a^	54.88 ± 11.86^a^	48.86 ± 13.57^b^	2.799	0.041
GLU (mmol/L)	7.18 ± 2.37^a^	7.71 ± 2.80^ac^	8.70 ± 3.20^bd^	8.53 ± 2.87^cd^	3.198	0.024
HBA_1-^c^_ (%)	8.56 ± 2.41	8.46 ± 1.97	8.25 ± 1.66	8.82 ± 1.68	0.440	0.725
ALT (mmol/L)	17.00 (13.00,25.00)^a^	20.00 (14.00,28.25)^ab^	25.50 (16.00,36.75)^b^	37.00 (24.60,67.50)^c^	36.963	<0.001
AST (mmol/L)	23.28 ± 18.79^a^	19.87 ± 6.59^a^	22.65 ± 10.93^a^	31.28 ± 12.79^b^	5.863	0.001
TC (mmol/L)	4.74±1.38^a^	4.60±1.08^a^	4.67±1.30^a^	5.46±1.45^b^	3.281	0.022
TG (mmol/L)	1.18 (0.86,1.68)^a^	1.89 (1.21,2.40)^b^	2.06 (1.53,3.13)^bc^	2.72 (1.74,3.32)^c^	43.805	<0.001
HDL-c (mmol/L)	1.15 ± 0.26^a^	1.05 ± 0.26^b^	0.99 ± 0.25^b^	1.03 ± 0.24^ab^	3.689	0.013
LDL-c (mmol/L)	3.03 ± 1.08	2.91 ± 0.82	3.06 ± 0.96	3.28 ± 0.76	1.070	0.363
UA (μmol/L)	310.49 ± 110.81^a^	335.45 ± 97.61^ab^	367.48 ± 109.68^bc^	400.92 ± 109.46^c^	5.422	0.001
SWE (m/s)	1.24 ± 0.12^a^	1.29 ± 0.15^b^	1.35 ± 0.09^c^	1.41 ± 0.13^c^	14.164	<0.001
ATI (dB**·**cm^-1^**·**MHz^-1^)	0.59 ± 0.07^a^	0.65 ± 0.07^b^	0.77 ± 0.09^c^	0.86 ± 0.08d	109.245	<0.001

**Note:** Different letters in the same row indicate significant differences between groups. BMI: body mass index, GLU: fasting blood glucose, HBA1-c: glycated haemoglobin, ALT: alanine aminotransferase, AST: aspartate aminotransferase, TC: total cholesterol, TG: triacylglycerol, HDL-c: high-density lipoprotein, LDL-c: low-density lipoprotein, UA: uric acid, ATI: acoustic tomography imaging, SWE: shear wave elasticit.

**Table 2:** Reproducibility of ATI and SWE for different operators.

-	**Operators**	**ICC**	**COV (%)**
ATI (dB·cm-1·MHz-1)	A	0.977 (0.912,0.999)	5.3
	B	0.995 (0.979,1.000)	3.4
SWE (m/s)	A	0.995 (0.980,1.000)	3.7
	B	0.998 (0.991,1.000)	2.9

**Table 3:** Correlation analysis between ATI and clinical data and fatty liver degree.

		**SWE**	**AGE**	**BMI**	**GLU (OH)**	**HBA1c**	**Grades of Fatty Liver**
ATI	r	0.363	-0.032	0.409	0.202	0.01	0.784
	P	<0.001	0.711	<0.001	0.029	0.913	<0.001
	-	ALT	AST	TP	ALB	GLB	SEX
ATI	r	0.290	0.113	0.122	0.164	0.037	-0.013
	P	0.001	0.198	0.176	0.067	0.685	0.882
	-	TC	TG	HDL-c	LDL-c	UA	-
ATI	r	0.156	0.456	-0.254	0.115	0.385	-
	P	0.079	<0.001	0.004	0.201	<0.001	-

**Note:** *F*=15.179,*P*<0.001,*R*^2^=0.254,*r*^2^=0.238.

**Table 4:** Regression coefficient estimation and ATI model test.

	**β**	**SE**	**B**	***t***	***p***	**95%CI**	
						**Lower**	**Upper**
constant	0.338	0.0745	-	4.535	<0.001	0.1909	0.4850
TG	0.014	0.0036	0.252	3.794	<0.001	0.0066	0.0208
BMI	0.052	0.0142	0.243	3.653	<0.001	0.0238	0.0797
ALT	0.001	0.0004	0.234	3.553	<0.001	0.0006	0.0020
SWE	0.113	0.0559	0.135	2.013	0.046	0.0022	0.2230

**Table 5:** Diagnostic efficacy of ATI in diagnosing different degrees of fatty liver.

**Grades of Fatty Liver**	**ATI Cut-off Value**	**AUC**	**Sensitivity**	**Specificity**	**Youden**
≥S1	0.665	0.899	0.706	1.000	0.706
≥S2	0.695	0.939	0.884	0.870	0.754
≥S3	0.745	0.937	0.966	0.808	0.773

## Data Availability

The datasets used and/or analysed during the current study will be available from the corresponding author [L.L] upon reasonable request.

## LIST OF ABBREVIATIONS

NAFLD: Non-alcoholic fatty liver disease
PDFF: Proton density fat fraction
SWE: Shear wave elastography
ALT: Alanine aminotransferase
TP: Total protein
GLB: Globulin
UA: Uric acid
LDL-C: Low-density lipoprotein cholesterol
HDL-C: High-density lipoprotein cholesterol
TG: Triglycerides
SD: Standard deviation
IQR: Interquartile range
VCTE: Vibration-controlled transient elastography
BSG: British Society of Gastroenterology
BASL: British Association for the Study of the Liver

## STANDARD OF REPORTING

STROBE guidelines were followed.
