# Supplementary material for: Correlation between Liver fat Content Determined by Ultrasonic Attenuation Imaging and Lipid Metabolism in Patients with Non-Alcoholic Fatty Liver Disease
Source: Curr Med Imaging. 2025 Mar 17;21:e15734056335310. doi: 10.2174/0115734056335310250217064323 (PMC13181203; doi:10.2174/0115734056335310250217064323)
Supplement: Supplementary file 1 [file CMIM-21-E15734056335310_SD1.pdf]

1573-4056/25

Send Orders for Reprints to reprints@benthamscience.net

BENTHAM SCIENCE

CrossMark

Current Medical Imaging

Content list available at: https://benthamscience.com/journals/cmir

Supplementary Material

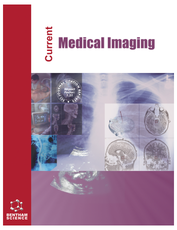

Correlation between Liver fat Content Determined by Ultrasonic Attenuation Imaging and Lipid Metabolism in Patients with Non-Alcoholic Fatty Liver Disease

Yanhong Hao[1], Yanjing Zhang[2], Guolin Yin[2], Lei Zhang[3] and Liping Liu[2,*]

[1]*Department of Ultrasonography, First Hospital of Shanxi Medical University, Taiyuan, Shanxi, 030001, China*
[2]*Department of Interventional Ultrasound, First Hospital of Shanxi Medical University, Taiyuan, Shanxi, 030001, China*
[3]*Department of Magnetic Resonance, First Hospital of Shanxi Medical University, Taiyuan, Shanxi, 030001, China*

Table S1. Comparison of SWE and ATI between different stratifications of the diabetic liver ultrasound normal group and the healthy control group.

Stratification (n)		SWE (m/s)	ATI (dB·cm^{-1}·MHz^{-1})
S0	HBA$_{1\text{-}c}$ < 7 (13)	1.30 ± 0.17a	0.58 ± 0.07ab
	HBA$_{1\text{-}c}$ = 7-9 (27)	1.22 ± 0.10a	0.61 ± 0.07b
	HBA$_{1\text{-}c}$ > 9 (20)	1.28 ± 0.16a	0.59 ± 0.08ab
control group (50)		1.14 ± 0.06b	0.55 ± 0.06a

Note: For pairwise comparisons between groups, different letters in the same column indicate significant differences.

Table S2. Diabetic liver ultrasound with different fatty liver grades compared with the healthy control group.

	Control	S0	S1	S2	S3
ATI (dB·cm-1·MHz-1)	0.55 ± 0.06	0.59 ± 0.07*	0.65 ± 0.07***	0.77 ± 0.09***	0.86 ± 0.08***
SWE (m/s)	1.14 ± 0.06	1.24 ± 0.12***	1.29 ± 0.15***	1.35 ± 0.09***	1.41 ± 0.13***

*: Compared to the control group, there was a statistically significant difference, $p < 0.05$.
***: Compared to the control group, there was a statistically significant difference, $p < 0.01$.

© 2025 The Author(s). Published by Bentham Science Publisher.

This is an open access article distributed under the terms of the Creative Commons Attribution 4.0 International Public License (CC-BY 4.0), a copy of which is available at: https://creativecommons.org/licenses/by/4.0/legalcode. This license permits unrestricted use, distribution, and reproduction in any medium, provided the original author and source are credited.

DOI: 10.2174/0115734056335310250217064323, 2025, *21*, e15734056335310
